# Does Guided Written Disclosure Reduce Distress and Improve Psychological Functioning in Patients with Skin Diseases?

**DOI:** 10.3390/ijerph19052943

**Published:** 2022-03-03

**Authors:** Rossella Mattea Quinto, Luca Iani, Francesco De Vincenzo, Francesca Russo, Piero Porcelli, Damiano Abeni

**Affiliations:** 1 Department of Human Sciences, European University of Rome, 00163 Rome, Italy; francesco.devincenzo@unier.it (F.D.V.); fraru2011@hotmail.it (F.R.); 2 Department of Psychological, Health and Territorial Sciences, University of “G. D’Annunzio” Chieti–Pescara, 66100 Chieti, Italy; piero.porcelli@unich.it; 3 Clinical Epidemiology Unit, Istituto Dermopatico dell’Immacolata, IDI-IRCCS, 00167 Rome, Italy; d.abeni@idi.it

**Keywords:** dermatology, expressive writing, sense of coherence, psychological distress, emotion regulation, skin-related quality of life, sense of peace, positive psychology, randomized controlled trial

## Abstract

**Background.** Skin diseases (e.g., psoriasis and systemic sclerosis) are generally associated with negative psychosocial outcomes. Although different psychological interventions have been used to improve the quality of life of dermatological patients, the effects of the guided written disclosure (GWD) protocol have not been previously examined in these patients. Moreover, little attention has been paid to positive psychology constructs. **Methods.** This study investigates the effectiveness of GWD on positive and negative functioning in dermatological patients. Pre- and 1-month post-intervention measures included emotion regulation, sense of inner peace, skin-related symptoms and functioning, sense of coherence, and psychological distress. **Results.** A total of 196 consecutive outpatients were randomly assigned to GWD and active control groups, of whom 60 (30.6%) completed the study and 45 (GWD: *n* = 24; AC: *n* = 21) provided complete data. Our results did not show any significant difference between the experimental and control groups in the outcome variables, whereas non-completers reported higher levels of distress, unpleasant skin-related emotions, and lower cognitive reappraisal compared to completers. **Conclusions.** These findings show a poor compliance, and suggest that expressive writing is not well accepted by patients and is not effective in improving positive and negative psychological functioning in dermatological patients.

## 1. Introduction

Skin diseases, such as psoriasis and systemic sclerosis, are associated with negative psychosocial outcomes, including low psychological well-being and high psychological distress [1], psychiatric morbidity [2], alexithymia and emotion dysregulation [3], quality of life (QoL) impairment [4], and low levels of spiritual well-being (i.e., meaning and sense dimensions) [5]. Various psychological interventions were aimed at reducing disease severity, skin-related symptoms, and different types of psychosocial distress, as well as improving health-related quality of life in dermatological patients [6,7]. However, researchers paid little or no attention to clinically relevant constructs based on the positive psychology framework [7], even though previous observational studies have suggested the importance of focusing on positive resources (e.g., sense of coherence [8,9], adaptive emotion regulation and positivity [10], and spiritual well-being [11]). For example, Iani et al. [10] found that higher cognitive reappraisal, sense of coherence, and positivity predicted higher spiritual well-being and lower psychological distress in patients with psoriasis and systemic sclerosis after controlling for negative functioning (i.e., physical symptoms, type of disease, and expressive suppression). These findings suggest that clinicians may facilitate spiritual well-being and reduce the distress associated with skin disease by promoting psychological resources [12].

The Guided Written Disclosure (GWD) protocol is a brief, cost-effective, and potentially promising intervention for dermatological patients. This intervention consists of writing at least 20 min a day for three sessions about traumatic or stressful life events. Patients are guided to describe (1) the stressful event without expressing emotions; (2) their thoughts and feelings at the time of the event as well as its impact on their life; and (3) how they currently think and feel about the event, whether their experience contributed to their personal growth, and how they will cope with similar events in the future [13,14]. The GWD is deemed to facilitate “cognitive coherence of one’s life situation” [13] (p. 373) and its writing instructions include the principles of cognitive reappraisal.

Previous research on the effects of GWD on health outcomes led to mixed results and the effectiveness of writing interventions is still unclear and controversial [14]. Gidron et al. [15] found that frequent clinic attenders in the GWD group made fewer clinic visits and reported lower levels of symptoms associated with somatization compared to those in the control group. Martino et al. found that patients with breast cancer following GWD reported a significant reduction in intrusive thoughts and irritability compared to the control group [16]. The same research group found that GWD was effective in reducing both symptoms (e.g., anxiety, depression) and mood states (tension-anxiety and fatigue-inertia) after treatment in parents of children with leukemia, especially in those with good emotional processing skills [17]. In contrast, the levels of distress worsened over time in the control group. Moreover, Arden-Close et al. [18] found that GWD had no effect on perceived stress, quality of life, intrusive thoughts, or couple communication about the disease in ovarian cancer patients and their partners. However, an increase in intrusive thoughts in the experimental group was not associated with an increase in perceived stress at the 3-month follow-up. The authors suggested that the writing exercise helped patients to deal with intrusive thoughts related to cancer.

Although GWD has never been investigated in dermatological patients, studies using a similar intervention, such as Pennebaker’s expressive writing, provided useful information. Indeed, Tabolli et al. [19] examined the effect of expressive writing on psychological distress and quality of life in patients with skin diseases. They found that the intervention had no effect when compared to controls. Similarly, another study with psoriasis patients undergoing phototherapy found that psychological distress, skin-related symptoms, and skin-related functioning did not significantly change after expressive writing, even if a longer remission was observed [20]. Both Pennebaker’s expressive writing and GWD share similar theoretical underpinnings; however, a key difference between them is the intervention format. Though participants are invited to write freely about their deepest thoughts and feelings in Pennebaker’s expressive writing, GWD has a structured format providing guidance on how and what to disclose of adverse or traumatic events [15]. This guidance is meant to organize the chronological description of events and to facilitate the reprocessing of memories related to stressful events into existing schemas. Thus, GWD is not merely focused on ventilation and re-experience as in Pennebaker’s expressive writing, but also guides individuals in the description of adverse events to enhance cognitive processing, and facilitates self-reflection of the past, present, and future effects of such events [18,21].

Null findings in previous studies using Pennebaker’s expressive writing with dermatological patients [19,20] may suggest that emotional expression is not sufficient to improve psychological outcomes. We propose that additional characteristics of GWD, including guidance in cognitive reprocessing, might be more beneficial for these patients. Overall, GWD has some advantages in medical contexts since it is brief and easy to implement. Previous research [3,22] reported the need to include psychological treatments in the medical management of individuals with psoriasis. Moreover, very few psychological interventions have been conducted with patients with systemic sclerosis and, to our knowledge, this is the first study on the effects of expressive writing involving this population. Therefore, because of the high potential public health implications of identifying a cost-effective intervention, we aimed to investigate the effects of GWD on positive and negative psychological functioning in dermatological patients. We expect that this intervention would promote a more adaptive psychological functioning (i.e., higher cognitive reappraisal, sense of peace, comprehensibility, and meaningfulness of illness experience) and reduce negative functioning (i.e., expressive suppression, skin-related symptoms, poor functioning, and psychological distress) in patients with psoriasis and systemic sclerosis.

## 2. Materials and Methods

### 2.1. Participant Characteristics

The present clinical trial involved 196 patients with skin diseases enrolled at the Istituto Dermopatico dell’Immacolata (IDI-IRCCS, Rome, Italy), a large tertiary care hospital and a reference dermatological center located in Rome, Italy, during the period from July 2017 to December 2019. Inclusion criteria were an age of 18 years or older, a diagnosis of psoriasis or systemic sclerosis, and the ability to complete questionnaires. Exclusion criteria were mental disorders and having received psychotherapy and/or psychopharmacological treatment for at least 6 months in the last 3 years. Clinical records were used to ascertain whether participants met inclusion and exclusion criteria. The trial was registered with https://www.clinicaltrials.gov (NCT04739228).

### 2.2. Measures

All participants were administered with a battery of psychological questionnaires. The internal consistency (Cronbach’s alphas) was satisfactory for all scales, ranging from alpha = 0.68 to alpha = 0.88. Data on clinical (e.g., symptoms, duration of the disease, comorbidities, etc.) and demographic characteristics were retrieved from medical records.

Skin-related functioning and emotions were assessed using the Skindex-29 [23]. The questions refer to the previous 4-week period, and scores are given on a 5-point frequency scale (0 = *never*; 4 = *all the time*). Sample items are “My skin condition makes it hard to work or practice hobbies” (functioning subscale) and “My skin condition makes me feel depressed” (emotion subscale). Higher scores indicate worse skin-related quality of life.

The short form of the Sense of Coherence Scale (SOC-13) [24] was used to measure two components of human experience: a cognitive component (i.e., comprehensibility/manageability) and a motivational component (i.e., meaningfulness). The questionnaire contains 13 items based on a 7-point Likert response scale. Sample items are “Do you have very mixed-up feelings and ideas?” (comprehensibility/manageability) and “Do you have the feeling that you really don’t care about what is going on around you?” (reverse item, meaningfulness). Higher scores indicate greater sense of coherence.

The 4-item Peace subscale of the Functional Assessment of Chronic Illness Therapy-Spiritual Well-Being Scale (FACIT-Sp) was used to assess the sense of inner peace [25]. Items are measured on a 5-point frequency scale (0 = *not at all*; 4 = *very much*). A sample item is “I feel peaceful”. Higher scores indicate greater sense of peace.

Emotional regulation was measured with the Emotion Regulation Questionnaire (ERQ) [26]. It was developed to assess individual differences in cognitive reappraisal and expressive suppression. The questionnaire contains 10 items based on a 7-point Likert scale (1 = *strongly disagree*; 7 = *strongly agree*). Sample items are “I control my emotions by changing the way I think about the situation I’m in” (cognitive reappraisal) and “I keep my emotions to myself” (expressive suppression).

Psychological distress was assessed with the 12-item General Health Questionnaire (GHQ-12) [27]. It contains items based on a 4-point scale; a sample item is “Have you recently felt constantly under strain”. Higher scores indicate greater psychological distress.

### 2.3. Experimental Design

A researcher not involved in the recruitment process assigned participants to the intervention group (the GWD group) and to the control group (the active control group) using computer-generated random numbers. Both groups received written instructions regarding the timing of the writing exercise (20 min, once a week for three weeks). Writing sessions were carried out alone at home; the first session was performed one week after enrollment. All participants were contacted by telephone by a research assistant the day before each writing session to recall the task. Measures were administered at pretest (T0) and after one-month (T1). The blinding of outcome assessment was not possible in this study. Patients were told to follow specific instructions according to treatment assignment (i.e., GWD or active control). However, they were not aware whether their writing task referred to treatment or active control group.

GWD Group. In the first session, patients were asked to write in a chronologically manner about the occurrence of the illness without expressing emotions. In the second session, patients were asked to write about their thoughts and feelings during the illness experience, and to what extent the illness affected their life. In the third session, patients were asked to write how they currently thought and felt about the illness, how they will deal with similar events in the future, and whether the illness experience contributed to their personal growth.

Active control group. In all three writing sessions, patients were asked to report daily events during the last week, without focusing on the emotional aspects related to the illness.

### 2.4. Statistical Analysis

A meta-analysis showed that guided written disclosure had an effect size of 0.89 on psychosocial outcomes [28]. Power analysis showed that with an alpha of 0.05 and a power of 0.80, we needed a sample of 34 participants to detect effect sizes of 0.89 and higher.

All statistical analyses were performed using SPSS (Version 19.0; IBM Corp, Armonk, NY, USA). Differences between GWD and control groups with respect to age, sex, and clinical variables were evaluated with *t* and χ^2^ tests. We conducted a 2 (group) × 2 (time [pre-treatment vs. post-treatment]) repeated measures multivariate analysis of variance (MANOVA) for a set of variables (i.e., skin-related emotions and functioning, cognitive reappraisal, expressive suppression, comprehensibility/manageability, meaningfulness, peace, and psychological distress). We also conducted a MANOVA to examine differences in psychological variables between completers and non-completers. The Shapiro–Wilk test was performed to assess the normality of distribution. All statistics were considered significant at *p* < 0.05. Occasional missing values were imputed by calculating the mean score of the subscale for each participant, and then replaced.

## 3. Results

### 3.1. Demographic and Clinical Characteristics

**Figure 1:**
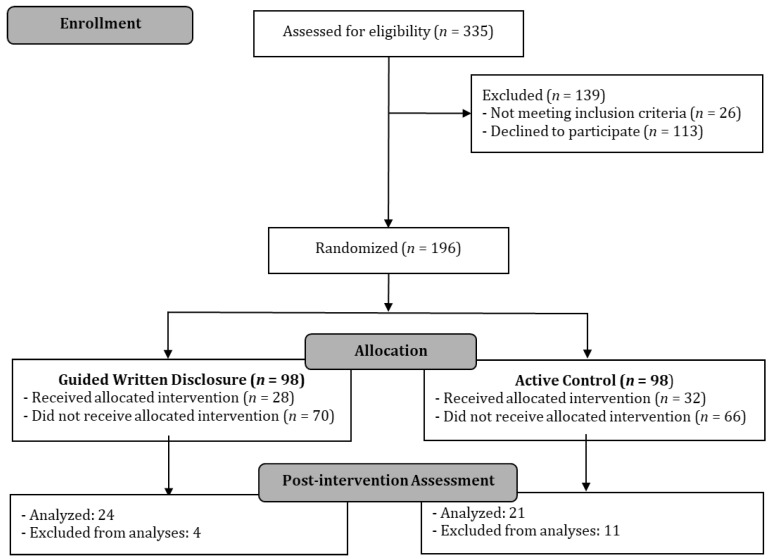
Flow chart of participants.

We assessed 335 patients for eligibility. Of these, 26 (7.8%) did not meet the inclusion criteria and 113 (33.7%) declined to participate. The main reasons for declining were lack of time, not being interested in the study, or inability to complete questionnaires. A total of 196 patients were randomized. Following randomization, 136 (69.4%) dropped out after the baseline assessment. Thus, 60 patients completed the study (see Figure 1). Fifteen completers (*n* = 25%) were excluded from the analyses because of missing data, thus leaving 45 patients for the analysis (*n* = 24 in the GWD group and *n* = 21 in the control groups).

Demographic and clinical characteristics of participants are reported in Table 1. The sample consisted of 45 patients (25 males and 20 females) with a mean age of 50.8 years (*SD* = 15.2; median = 10.0 months). More than two-thirds of participants were diagnosed with psoriasis (68.9%), while 31.1% had a diagnosis of systemic sclerosis. The mean disease duration was 13.9 months. At diagnosis, 73.8% of patients (*n* = 31) reported zero or one physical comorbidities, whereas 26.2% of patients (*n* = 11) reported two or more comorbidities. There were no between-group differences on demographic, clinical, and psychological characteristics at baseline assessment (Table 1). The Shapiro–Wilk test showed that the data were normally distributed, except for cognitive reappraisal (*p* = 0.049) and psychological distress (*p* = 0.039) at pretest, and skin-related functioning (*p* = 0.044) at posttest. However, even if normality is violated, MANOVA procedures are robust enough for type I error [29].

### 3.2. Effectiveness of GWD

Table 2 shows means and standard deviations of outcome measures at pre- and post-test. The MANOVA showed no significant group x time interaction in any variable, *F* (8, 35) = 0.126, *p* = 0.998, *η*_p_^2^ = 0.03.

### 3.3. Attrition

About 16% of patients were excluded from the analyses due to missing values (*n* = 31). Treatment completers and non-completers did not differ in sociodemographic and clinical characteristics (i.e., age, gender, type of disease, disease duration, and comorbidities). However, non-completers reported higher levels of psychological distress and skin-related negative emotions, as well as lower levels of cognitive reappraisal, compared to completers (see Table 3).

## 4. Discussion

The aim of the present study was to examine the effects of GWD in reducing psychological distress and expressive suppression, and improving peace, sense of coherence, cognitive reappraisal, and skin-related quality of life in patients with psoriasis and systemic sclerosis. Our results did not show any significant difference between the experimental and control groups in the outcome variables. Regarding psychological distress and skin-related quality of life, the findings of this study are similar to those of previous research that investigated the effects of the Pennebaker writing paradigm in dermatological patients. For example, Paradisi et al. [20] found no significant differences between pre- and post-expressive writing in psychological distress, skin-related symptoms, and skin-related functioning in patients with psoriasis treated with phototherapy, although they had longer psoriasis’ remission after phototherapy. Moreover, expressive writing did not improve skin-related quality of life and psychological distress in patients with psoriasis [19]. Whereas Pennebaker’s expressive writing is focused on expression of emotions, GWD additionally enhances a cognitive reprocessing by means of structured guidance [15,18,21]. Hence, null findings in our study suggest that additional characteristics of GWD do not provide benefits to dermatological patients. Furthermore, expressive writing did not have a beneficial effect in improving depression, anxiety, and distress in patients with advanced cancer [30]. Finally, expressive writing improved physical but not psychological health outcomes in different clinical populations [31]. Conversely, a meta-analysis showed that written disclosure was effective in improving distress in different populations [14]. It is worth noting, however, that none of these studies were conducted on patients with skin diseases. Therefore, our results and those of previous studies on dermatological patients [19,20] seem to suggest that different expressive writing interventions are not beneficial for these patients.

In our study, GWD did not improve cognitive reappraisal or expressive suppression. To the best of our knowledge, no previous studies have examined the effects of GWD or expressive writing on emotion regulation in dermatological patients. It has been suggested that expressive writing has particular benefits for individuals with emotion dysregulation [32], especially to facilitate reappraisal and awareness of emotions [33]. For example, Suhr et al. [34] found that positive writing significantly improved expressive suppression and cognitive reappraisal in individuals discharged from psychiatric inpatient treatments, suggesting that writing tasks promote emotional self-regulation through describing positive and negative feelings. In light of these findings, we hypothesize that GWD had no effects on emotion regulation because of psychological features of dermatological conditions. Indeed, patients with skin diseases are characterized by high levels of alexithymia [35,36,37] that may have hindered improvements in emotion regulation associated with writing tasks.

In our study, we also hypothesized that writing about stressful events would have positive effects on the sense of coherence and sense of peace. Previous studies showed that different narrative interventions with patients with severe health conditions were effective in maintaining or increasing the sense of peace [38,39]. The use of narrative interventions designed to promote meaning and hope or to highlight mastery and values can help patients with chronic diseases restore a sense of peace. However, we found that GWD had no effects on the sense of peace. It is likely that this result may be due to clinical features of psoriasis and systemic sclerosis, characterized by unpredictability of psoriasis or worsening of systemic sclerosis symptoms that may hinder the increase in inner peace. Moreover, dermatological patients often live in constant fear of recurrence [40] [Rakhesh et al., 2008]. This awareness may have inhibited the sense of inner peace after participating in a guided written disclosure protocol.

Mez and Jemec [41] reported that patients with psoriasis have a low sense of coherence, which can lead to insufficient appraisal of the illness experience. Mura et al. [42] suggested that catastrophizing and a low sense of coherence, being associated with depressive thoughts and lower quality of life in patients with systemic sclerosis, could be modified, and these patients could also benefit from training in more functional coping styles. Indeed, the enhancement of the sense of coherence may be a prerequisite for adequate appraisal in patients with psoriasis [41]. Different psychological interventions have been implemented to increase the sense of coherence [43,44]; however, little is known about the effects of expressive writing for improving sense of coherence in patients with skin diseases. Boals [45] posited that meaning-making, which appears to be a healthy and adaptive type of cognitive processing, may be a possible mechanism involved in the positive effects of expressive writing interventions. In his study, he found that an increase in meaning-making was associated with a greater decrease in intrusive thoughts in participants who wrote about a highly stressful event during an expressive writing intervention. The results of Boals’ study suggest that expressive writing can be effective because it gives individuals an opportunity to engage in the meaning-making process, especially for individuals who are particularly distressed by negative events. In line with these findings, it is likely that GWD did not improve the sense of coherence because patients were not highly distressed by their condition. Indeed, non-completers not only reported lower cognitive reappraisal, but also higher psychological distress and more negative emotions associated with skin diseases compared to completers. It is likely that the characteristics of patients who completed the study (e.g., being less distressed) may partly explain the ineffectiveness of GWD in promoting a sense of coherence.

Finally, our attrition rates were poorer than expected and higher than those reported in previous studies investigating emotional disclosure interventions in psoriasis management [6]. Indeed, about 70% of eligible patients did not complete the protocol and showed very low interest in participating in the GWD. However, attrition rates of dermatological patients during expressive writing interventions are a significant problem, ranging from 14.5% to 54.9% [6]. The high dropout rate may indicate that more emotionally dysregulated patients are less likely to benefit from GWD as they have a higher chance of dropping out. In this vein, our results revealed that emotional and psychological distress are the most important predictors of attrition from GWD. The very high attrition rate in our study could have introduced bias; therefore, these results should be viewed with caution. Although the current results cannot be generalized to clinical populations, they suggest that the use of GWD is problematic in dermatology settings. Thus, due to very high levels of attrition, the feasibility of GWD for patients with skin diseases seems uncertain.

This study has several limitations. Firstly, the small sample size has reduced statistical power. Moreover, we did not carry out separate analyses for each group. Secondly, a longer follow-up was not available due to patients’ poor compliance. Thirdly, there was a high level of attrition.

Despite the null findings in the present study, there is a need for psychological interventions aimed at improving spiritual well-being, sense of coherence, and emotion regulation in dermatological patients. Little is known about therapeutic approaches that consider these aspects of human experience in dermatology, since previous studies have mainly focused on negative functioning. However, positive aspects of human functioning may be relevant to improve well-being and reduce psychological distress in these patients. For example, a mindfulness-based cognitive therapy group therapy was effective in improving quality of life in patients with psoriasis [46]. Moreover, compassion-based and mindfulness-based self-help were found to be acceptable in people with psoriasis, and both interventions showed reductions in shame and improvements in quality of life [47]. A compassion-focused self-help has also shown promise in reducing perceived stress, anxiety, depression and impairment of quality of life, as well in improving self-compassion, in patients with skin conditions [48]. Thus, mindfulness- and compassion-based practices have potential for use as brief interventions in dermatology settings, and warrant further investigation in this context.

In summary, to our knowledge, this is the first study examining the effects of GWD on psychological functioning in patients with skin diseases. The strengths of the present study are that it is an RCT and patients are not aware of whether their writing task refers to the treatment or active control group. Another strength is that this is the first study with a structured format guiding dermatological patients in the description of adverse events to enhance cognitive reprocessing. A further strength is that this is the first study to examine the effects of an expressive writing intervention on positive functioning in dermatological patients. A final strength is that it highlights the psychological characteristics of patients who do not adhere to the guided expressive writing intervention, including high levels of psychological distress and skin-related negative emotions, as well as lower levels of cognitive reappraisal. Our findings suggest that the use of GWD is problematic in dermatology settings and that, due to very high levels of attrition, the feasibility of this protocol seems uncertain for patients with skin diseases.

## Figures and Tables

**Table 1:** Demographic and clinical variables according to treatment condition at baseline.

Variable, *n* (%)	GWD	AC	Total	*t* or X^2^	*p*
			Sample		
	(*N* = 24)	(*N* = 21)	(*N* = 45)		
Age, *M* (*SD*)	53.0 (13.9)	48.1 (16.5)	50.8 (15.2)	*t *(39.4) = 1.07	0.293
Sex				X^2^ (1, *N* = 45) = 1.16	0.688
Male	14 (58.3)	11 (52.4)	25 (55.6)		
Female	10 (41.7)	10 (47.6)	20 (44.4)		
Disease				X^2^ (1, *N* = 45) = 9.79	0.322
Psoriasis	15 (62.5)	16 (76.2)	31 (68.9)		
Systemic sclerosis	9 (37.5)	5 (23.8)	14 (31.1)		
Disease duration (months), *M* (*SD*)	15.7 (12.0)	13.8 (11.1)	13.9 (12.5)	*t *(36.1) = 0.92	0.364
Comorbidities				X^2^ (1, *N* = 42) = 1.94	0.163
≤1	15 (65.2)	16 (84.2)	31 (73.8)		
≥2	8 (34.8)	3 (15.8)	11 (26.2)		

GWD group = guided written disclosure group; AC group = active control group.

**Table 2:** Means and standard deviations of outcome variables in GWD and control groups.

Outcome Variables	GWD (*n* = 23)		AC (*n* = 21)	
	Pre-Test	Post-Test	Pre-Test	Post-Test
Skin-related emotions	14.9 (8.7)	14.8 (8.8)	15.7 (9.3)	15.5 (9.5)
Skin-related functioning	15.7 (10.6)	14.7 (9.9)	14.7 (10.6)	13.6 (10.1)
Cognitive reappraisal	29.7 (8.5)	28.6 (8.8)	29.5 (7.9)	28.4 (8.0)
Expressive suppression	15.0 (5.3)	14.3 (6.1)	15.2 (6.0)	13.8 (7.1)
Comprehensibility/manageability	34.6 (4.9)	35.0 (5.6)	33.5 (9.7)	35.3 (8.5)
Meaningfulness	20.4 (4.2)	20.5 (4.3)	19.3 (6.1)	19.4 (5.5)
Peace	8.4 (3.2)	9.1 (4.0)	8.8 (4.2)	9.5 (3.8)
Psychological distress	13.0 (6.0)	11.6 (5.5)	12.5 (5.7)	10.9 (5.8)

*Note*: Standard deviations are in parentheses. GWD group = guided written disclosure group; AC group = active control group.

**Table 3:** Means, standard deviations, and multivariate analyses of variance of outcome variables in completers and non-completers.

Variable	Completers	Non-Completers	*F*	*p*
	(*n* = 45)	(*n* = 120)		
Skin-related emotions	15.24 (8.85)	18.65 (9.14)	4.62	0.033
Skin-related functioning	15.42 (10.44)	18.59 (11.84)	2.48	0.117
Cognitive reappraisal	29.78 (8.15)	26.47 (8.54)	5.03	0.026
Expressive suppression	15.19 (5.56)	14.18 (6.32)	0.90	0.344
Comprehensibility/manageability	19.84 (5.18)	20.36 (4.84)	0.36	0.550
Meaningfulness	34.11 (7.44)	32.60 (8.09)	1.19	0.277
Peace	8.67 (3.70)	8.10 (3.35)	0.90	0.343
Psychological distress	12.95 (5.88)	15.50 (6.82)	4.89	0.028

*Note*. Standard deviations are in parentheses.

## Data Availability

The data presented in this study are available on request from the corresponding authors (R.M.Q., L.I.). The data are not publicly available due to privacy or ethical restrictions.
